# Molecular analysis of human T-cell lymphotropic virus type 1 and 2 (HTLV-1/2) seroindeterminate blood donors from Northeast of Iran; evidence of proviral tax, env, and gag sequences

**DOI:** 10.1186/1742-4690-8-S1-A66

**Published:** 2011-06-06

**Authors:** Delaram S Zanjani, Majid Shahabi

**Affiliations:** 1 Mashhad transfusion service, Research center of Iranian Blood Transfusion Organization, Mashhad, Iran; 2 Research center of Iranian Blood Transfusion Organization (IBTO), Tehran, Iran

## Background

Human T-cell lymphotropic virus type 1 and 2 (HTLV-1/2) Western blot indeterminate results are a problem of blood banks in endemic areas.

## Materials and methods

To determine the prevalence of HTLV-1/2 infection among indeterminate donors, we analysed 130 cases from Mashhad, an HTLV-1/2 endemic area in northeast of Iran.

## Results

The most frequent Western blot bands were GD21 alone (37.2%) followed by rgp46-2 alone (32.1%). We further tested 40 available DNA samples of these cases by PCR for viral sequences; tax, gag, and pol and found 5 cases (12.5%) to be positive for two or three HTLV-1 genes. There were no significant age, sex, and blood group differences between PCR positive and PCR negative cases. Among PCR positive individuals, the most prevalent Western blot bands were variable combinations of rgp46-1, GD21 and gp21 bands. The mean of optical density (OD) of enzyme linked immunosorbent assay (ELISA) test were was significantly higher in PCR positive individuals. The frequency of rgp46-1 band was also significantly higher in PCR positive cases, compared to PCR negative ones.

## Conclusions

The majority of HTLV-indeterminate donors lack HTLV provirus and therefore are not considered infected. However, in some cases with higher ODs in ELISA test and seroreactivity to env proteins; in particular rgp46-1 and GD21 may be indicative of infection and need further evaluation by molecular methods.

